# Supplementary material for: Obtaining Specific Sequence Tags for Yersinia pestis and Visually Detecting Them Using the CRISPR-Cas12a System
Source: Pathogens. 2021 May 6;10(5):562. doi: 10.3390/pathogens10050562 (PMC8148545; doi:10.3390/pathogens10050562)
Supplement: Supplementary file 1 [file pathogens-10-00562-s001.zip › pathogens-1175566-SI.pdf]

Table S1. PCR conditions

Reagent names	Volume (μL)
DEPC water	19
ExTaq	25
Primer A(10μmol/L)	2
Primer B(10μmol/L)	2
template DNA	2

Table S2. Cas12a reaction conditions

Reagent names	Volume (μL)
DEPC water	12
NEBuffer 3	2
Cas12a	1
Probe	2
crRNA(10μmol/L)	1
PCR/RPA product	2

Table S3. RPA reaction conditions

Reagent names	Volume (μL)
Primer A(10μmol/L)	2.4
Primer B(10μmol/L)	2.4
2×Reaction Buffer	25
dNTPs(10mM each)	2.3
10×Probe E-mix	5
20×Core Reaction Mix	2.5
DEPC water	6.9
template DNA	1
280mM MgOAc	2.5

Table S4. The 97 *Y. pestis*-specific tags obtained in this study*

Tag names	Start site	End site	Sequences (5′-3′)
YP_Etag 001	163534	163634	TTGTTCTGGATTATCGGTAACGTTCTCACTGTTTCAACAATGAATTCAATGGATTGTCTGATGATGATCAGCATTAAAAGGAAGTAAGCTGATGACTATT
YP_Etag 002	416199	416299	ACGATGCGGTAGCTGCTGGAAACCATGGTGCCGAGTAGCAACATCAGCATCATGAATAGCAGGTTGCTGGCATTCTGAATTATCAGCATAGCTGTCTTAT
YP_Etag 003	421710	421810	TAGCTGCTTTTATTCTGGTTGCCAGCCAGCGAGGGGAGGAAAGCCCAATGACCAAGCAAGTGACATTTATCTGCGGGCTTCTTAATGGCGTTCATGCCCG
YP_Etag 004	421811	421911	CCTGCGAGCCATATAGAAAGGGTATGCAACCGCTTCCAATCGCGTTTCCACTGGTATAATCCGCGTTCTGGCATCGTGGGAGATGGCAAGAGCGTACTTT
YP_Etag 005	423005	423155	AATCAAGGTGCCGAATCCATTGGCTTGTTTCGTACTGAAATATTGTATATGGATCGTGCAGCCGCGCCTTCAGAAGAGGAGCTTTATACCCTCTACGCTCAGGCACTTGGCGCTGCCAAGGGCAAACCGATTATTATTCGCACCATCGAT

YP_Etag 006	424204	424304	TTTTTCCAAACTGGCGCGTCGGATCATGCACGAGGGGTTCCGCGATGCGCTGGTTAGCGCTGGGACGGCCCAGCAAGTAGAAATGCTGCTGAAACACGAG
YP_Etag 007	467679	467829	TTTTGGTTAAAACATAATGGCAGTGGATTGAGTGCGGAACAGGTAAAAGTGCTTAATCGCCTATTAGATGGCGGTGAGAATGGCTTTTCTGCTGGGATAAATGCGTCACAATATCAGAAAGTCGCAAAGGTCAGTAAAGCAACCGCCACG
YP_Etag 008	508775	508875	TTTCTCGGCCCATGGTGTGTCACAGGCCGTTCGGGCAGAGGCCCGTTCACGTAATTTGACGATGCTGTTTGATGCAACCTGTCCATTGGTTACCAAAGTC
YP_Etag 009	517030	517180	AAAATGCGGCTTTGAGTGAGATGGGATGCTGTGCAGTATTATCAATACTAAACTTACTTTCTCCCCGTGACAGCAGTGCTTGAATACCAATCCAGATCAGGTAGCAGGCCCCGGCATATTTCAGCAAATTGAATAACCATGGTGTGGTCG
YP_Etag 010	533856	533956	GATCTTTCTGGCCACAGAGCAAAGCCTCTTCCTGCTGTTGAAGATCGTAAATTCCTTACTTTCGATCAGGATAATTTCGATGCCCGGATGAGAGCAATTA
YP_Etag 011	553573	553723	GCCGATATTGCGGTGTACTTGTTCCTCAACAATATCGATAAGTTACCTGGCGGCGAAACCTTCCTCTCGTTAATGAACGACGAACTTCTGGCCGGAGGGCTTGGGATTAGCATGACCCATAATGAAAAGGGGCAGAACAATTTCGTTGCC
YP_Etag 012	859300	859400	GAAATTTGAGTTCCAGAGGAAAATTTCCGGTGTTGGAAGCGGTACGCAAAACATAATCAATGGATACAGAAATACAGGGAGGTCTGCGATACGACTCGCA
YP_Etag 013	861387	861487	ATTCACTTGCCGTATGACTGCAACCTCGATGGCTTTTGGTATTTATAAGCATTCTACTTCCCACCCCGAATCAGCCCACCTAAACCACGGCGCTCCATAA
YP_Etag 014	1000314	1000464	GGGGGCGGCCTCTTGCCCTAACCGACGACCAAACCAGATCTCAGCGGTTAAATCCCGGATCCGGTTAAACAGCACGCTGTGGTTATGAGTCTCATCGCTTTTAACCAGAATCAACACCGCTTCTGGCTCGTTGAAGCCCGTCAGATAGCT
YP_Etag 015	1034125	1034225	GCCCGTCAGTTAGTGCAGCAAGCCGCTTCCGGGGCTTGTGCTCTGGTCGAGGCTAACCCACACGTGCCACTTTCGGCCTTGCGAGAGCAGGTCACGTCCA
YP_Etag 016	1056025	1056175	CAGCCGTCAGTGCTGCTGATAGCGATCAGCGGGAAAATATCGATAGCCGCGCGCTGTTCATTCAAGACTCGATGCGCCTAAACGAGCACTGGCTATTACTCGGTGGCCTGCGCTACGACAGCTTCGATGTGATGGCAGGTAAAGGCCGCC
YP_Etag 017	1568717	1568817	TGGTGCCCGAGCACAGTTTGCTGCGGTGCCATTGAACTTGCTTGGTGACCGCAACAGCGCTGACTATATCGATGGTGGCTATTGGGCGCACAGTGCGATT
YP_Etag 018	1569280	1569380	GCGAAATGGAAAAGCGCAATCAGGCGAAAGCTGAACTATTGTACGGTGCCATCGACCGCACAGGCTTCTATCGCAATCAGGTTGCTATTACTAACCGCTC
YP_Etag 019	1774064	1774214	GTCATTCAAGGTATTCGGGTAGTTACGGGCGCGGAAGCGGAATAACAGGGCCTCCAACCGTGGGTCCTGAAATGTCAAATCCAGTGCTGGCAGATTTTGTGACTCAGTTTGCTGGATAATCTTTATGGTGGCGCGATCAGCATCACTGAA
YP_Etag 020	1846828	1846928	GTTGCGCCCGATCACTTAAATCTTGCACCAACTTCAATTTGGTTGGCCCTTCCAAACGGTAAATCTGTGGTTGCCGTTGTAACAGCCCAATCAAATACAC

YP_Etag 021	1868734	1868834	TAGTTGTAGTGATATCACTATTATCTATCAATTATACGAGGCTATTGAGGTTCGATTGGTTTTGTTAAAGGTTTGTATATGATATGTGTTGTGTATTAAC
YP_Etag 022	2085369	2085469	TGGCTCTTTAATTGCGTCTGGATAGAACGCAATGTGCCATCCCCGACTAATGCACCATTACTCGTGGATTGATCCTGGCCTGTCTCTACAACGGTATATT
YP_Etag 023	2085954	2086054	TTTTGGCCGGGTTGAGTAATCGTGATCGTGCGTGTTGCATCACTGCTACCCAATTTATCTGTAGCGCTAGGCACATCTGCCGATAACAGCGATTGTGCTT
YP_Etag 024	2087369	2087469	AATATAAAGGTGAAGTGGATGCTACTAATGGAAAAGTAACATTTGCAGATGATGCTAATGGCGATCCAATTGATGACGCTACCAAGCTGGAAGCAGCGGC
YP_Etag 025	2363736	2363836	ACGAAAGGCGATCGCTCTATGTACCCATTCGCAACAGCGAACTCCATCATCGCGGCAAAACAAGAAAGGTAGACGTTTACCGTTCTGACAGACCGACCTT
YP_Etag 026	2482968	2483118	CAAAGGAGTAACGGTCAACTTTGGTTAAATCTGCAACATAGCGGCTACCGGGTTCGCAAATACGCGCTAGATCGTTACGAGCCAGATCCACCAACATTAAATGTTCTGCCATCTCTTTATGGTCAGTACGCATTTCCAACTCGATACGGC
YP_Etag 027	2493249	2493399	AAGAATACCCGCAAGATTCTGCGTAGCGGTGAAGTTGCACCACCGAAAGAAGATCCGGTACCATTGTTAGAGTTGCCTTGCGAGAAATCAGATGCTTATTTCGTCCTACGAGATGGCGCAGCTGGCGTGTTCTTGGCAGCCAATACATTC
YP_Etag 028	2847196	2847296	AGATCAACGGCATCAGTCCCGACACCAAATATTGAGATGATCTGCACTTCAGGCAATAACGCCAGGACCTCATTAGTGACGCCAATATCACCTCTAGTCA
YP_Etag 029	2847692	2847842	TTATCATTACCGAAGCTGAATTGTTTCAGCTACAGAATAGGCGTTAACAATCTCTGTCGCACCTTAGGGGGGAATGTTATTGGTTGATACGCATTGTTATACGTTGGCGTCACCTGTTAACAGGCGATAATTGATGAGCCGATGCTCTAC
YP_Etag 030	2855736	2855886	GGCTTCGCTATCCGCCGCCGGTGCGTACATAAACAAGCGCATCACTACGGCAAAGATAGCAATCTTGCTGGCGGTCGCCAGGAACGTGGAAACTGGCGCGGGTGCGCCCTGATACACATCGGGTGTCCACAATTGGAACGGTACCAACGA
YP_Etag 031	2936317	2936417	CCTATTCCTGAAAGGATTCGGTTGGGCACCGTTCTTAAAATACCAAATAGTGAACCGACGAAGAAGGCGATCACCCAAGCACATAATGATAATGCGACCG
YP_Etag 032	2994868	2994968	CCCCCTTGATGAACCTCATGGCTCTGGTCTGCACATTCACGGTATTCACTCCCACGAAGAAGGCCATAGTCATGGTGATCACGATCATGACCACAGCCAT
YP_Etag 033	3196040	3196140	TCGGGACCAGTCGCTGGCTGACAGCACGGCAACGCCAGAGTGAACCAATCAATAAACCCACCTGCAATTTTCATCATTTTCATTACCACCATTGGTGAAA
YP_Etag 034	3292861	3293011	CCCCCTCAATGGGCGTGATCCAACTTAAGCCAAATCTGAAGGAAGGAGATTTGACTCAAGGGTATTGTGTTCCCAGGGGCAGCTCGTTCTTCACCCATATCGCACCTGGCGAGACAACCCGCTGCGAGTTCCGCAGTGGTCAGGATGTGG
YP_Etag 035	3293888	3294038	GGCTCCTTATGGCGATAGCTTGCGCTACCTCTCTATTGAAGCTCAGGTGACTAATCGGGATCTTCCGCGCCTGATTTCCGCCAGTGGTAATTTTGAATTGACCATGTCGGATTCAGCGCCCGTTCACGGTGCCCGGTTTGTCTTCCAACC

YP_Etag 036	3296504	3296604	CCCCATCCCCACAGTTGCAGCCGTTACAGTTGCAATCGGCACAGTTGCAACCTGTACCGATACAGTCACAGCCGGTGCCTGTCCAGCAGCAAGGGATCCA
YP_Etag 037	3297227	3297347	CGTATCCTGCAACTTCACACTTCAGATTCGAACTCTATAAAATTAAAGATACGTTGAATTTAACTGATACCAACGGCGAACGTGTTCTGCCGGGTGGCACAATAGCTTATACCTGGGCGA
YP_Etag 038	3297578	3297678	CCGATAATAATTACATCAAGGTAAAAGACAGCCAGAACCAAGAAGACCGGTTGCTTATCAAAATCAGCGACACCAACGGTCAACAGATGAAAGTGAATGG
YP_Etag 039	3300059	3300159	CCCCCAAGTGTCGACTCCCATTCCGCAGGAGACAGCGGAACCTGTAGAACAGGAGAGTACGGTTATTTTAATTCTCGGTCCCTATGCTGAGAAATGGTTT
YP_Etag 040	3300389	3300539	CCCCCTCCCCTGCGTATTTGCTATTTACGCGCAGTTGAGTAATGAACGCCTTAGCCATAATTCTGATAACGCATATTGGACTGGAAATATTAATCTTGCGAACCAGCAGCCTATCGAGATTACGCAAGCCCTACAGTCACTGAGCCAACG
YP_Etag 041	3300529	3300629	TGAGCCAACGACTTGAGTTACAAGACATCAATCACTCAAGATTTGCAACTCAGCGTAATGTTATGGCGCATAATTTATTTACTTGGTTGAATGAATCGGG
YP_Etag 042	3306447	3306597	ATCTCTGGCGCGGGAGTATTGGCTTCCAGCAAACATCCGCAGCAAGCCCAGAAATTCGTGCAATGGGTCACCGGCAAAGCGGGGCAGGATTTCTTGCGCGAGAGCAATAGCGCCTTTGAATATGCGGTTGGCGTGAATGCCGCTTCCAAT
YP_Etag 043	3365861	3366001	TTGATTACCTTTGAATACCCTTGGCCCTTGACGTTGCAACGGTGTTCACCGCTCTCATGAACCCGAATGACTGACGCCTGTCAGCTCATCGGGGTTTGCGCGTTAGCGGCCTTGCTGCAACAACAATGACTTGGGTCGGT
YP_Etag 044	3398458	3398608	TAGTCCGCCAGCAATTTCAGGTTTAGGTGGGTCGTCAGGTTTCGATATGGAATTACAGGACCATGGCGGCCATGGCCATGATAAATTGATGGTCGCCCGCAACCAATTGTTGCAGATGGCGTCGCAGGAACCCGCGTTAACCCGAGTGCG
YP_Etag 045	3415596	3415696	GCACAAGCGTGCCAATCAGAGAAGAATAGATCTTATGATTATCGATTAACGGCTCAGCTAAACCTAGTGACTAGCAAACGTGTTACAGCTATTAGGATCA
YP_Etag 046	3432506	3432606	AAAGCAGTTACTCGATAGCTTGGGTTTGGCAACTGCGCCGTGGCAATTGTTATCCAGCGCCAGTGAATGGCCTGAGGTGTTCGCCACGTTGGGTGAGCTA
YP_Etag 047	3433765	3433865	ACCAACCTGTTTCAACATCGCACCGATGTCTAACAAACTCAGTGTTGGGAACTGGCGATTTAACTGAACCAAAGCCTCTCCATCACCGTGATAGCGGAAG
YP_Etag 048	3434740	3434890	CCCCACCAGCAACGAAATCAATACTAATGATCCCATTGACCACACCCATGGCCATAGCCCGGCAGTGGGTAACTCTGCGGGCAATACCGGCGCTAATAACCAAACCAATAGCCTCTCAAATAAGAGACCAATGACACTGCCGCAGAGGCC
YP_Etag 049(YP-1)	3580080	3580180	TTTCCAAATAATTATGTAGGTGACAATTGTATACCTGCATAATTAATTAGCAATCAACCAGACTCGCTCCACACATTACATACATTACCAATCAACCCCC
YP_Etag 050	3580236	3580336	ACCAGTGACGGGGTTTGCGTTCCATCAAGTACATGATGGGCGACGTCCTGATACAACGCTTCACGACTAGCCAACACATCCAGTATTTCTTCAACGATGG
YP_Etag 051	3614292	3614392	TTAAGGAAGTTATTTCGCTCGAATCGCAATGGGTGCGAGCACCCATTTCCGGCTTCATTAAGGTTAGTGATCACATGATCGGCATGGCCAAAAGTCGATG

YP_Etag 052	3614656	3614756	GCAATGAAAACACAACTGGTGCCTCTACCACGGGTGTTAATTCAACTGACTTATCCGCCATTACCCGATCTCCCTATCAGCTCAGGGCTTACCCTCTATT
YP_Etag 053(YP-2)	3615675	3615825	TTTACCCACGTCGATGCCGGTGCCATGGACCGTGTCGCCATGCACCACGCTGCCATGGTTACCGGACAGATTGGCCGCCAGATCCACGCCCTTGCCCTCGGTACTGTTGCCATTGAGCGTAGCGTTATCCAGCGTGCTCTCGCCATCGAT
YP_Etag 054	3616110	3616230	AGTGCCTGTTGTCGCCACACCTTGTAGAATCAACAAGTCAGCTTCACTGGTCCCGGTAACGTTTGAATTTTCGCCAATATCAATACCGTAGCCCGTGCCAGTAGTCGTTCCGTTAATCGA
YP_Etag 055	3747405	3747505	GCTGGGTATCCCAAATGACGCCCTTCCATCGCCGCTGCTACGGTACCCGAATAGATAACATCATCCCCTAAATTAGGGCCAGCATTAATACCAGAGACAA
YP_Etag 056	3773215	3773365	GCACTACCAAGCCAATCACAAGTAGAAGCCGAGCAACTTAAACAAATCGGCGCCAAAGATTGTCTGCTGAAACCCATATCGTCTATCAGGCTTATCCCTTTACTCTTGGCCGAAGACACGCACAGTAAAGTACCATCAGAGGAGCAACCA
YP_Etag 057	3794158	3794258	TAACGCCAACTTGCCACCCTTTAGCGCCAACTTACTGTCAGTGAACTCAGATTCGCGGTCAATTAGCCCTAATTCGCCACCGACTCACGCCAAATCGCTG
YP_Etag 058	3800871	3801021	ATTTTGATGGCCGCCTGGTTGGGTAAAGCTCGTCTGATTGATAACCAACTGGTTGATTTGCGCCACTGATATTTTACCGATCAGGCGGTTGCTCATTCCCCTCAAGCCGCGAGTTGGCCCAAAATCAACCAGGAACGCCAAGGTAAAAGT
YP_Etag 059	3853167	3853267	GGGGAAGCCAGATTGGGCATAAAGAATCAATCAAGGATACCGCCCGTGTATTAGGCCGTATGTATGACGGCATTCAATATCGTGGCTATGGGCAACGCGT
YP_Etag 060	3853273	3853423	AACACTCGCAGAATTTGCCGGCGTACCGGTCTGGAATGGCCTGACCGATGAGTTTCATCCAACCCAACTCCTGGCTGATTTGCTGACTATGCGGGAACACCTGCCCAATAAATCGCTGAATAAAATGACGTTGGCGTACCTCGGTGATAC
YP_Etag 061	3853453	3853553	AGCGGCCGCACTGGTAGGAATGGATTTACGCCTAGTCGCACCGAAAGCCTGTTGGCCAGAGGAAGCCTTCGTTATCAGTTGCCAGGCACTGGCACAGAAA
YP_Etag 062	3943702	3943802	AAAAGGGTCAGGTCATCCCGCAAGCCGACAACAATTGGCGTCTGGCAATTCTTGATAGCAAGCAACCTTTGGATATTCGTTTTGAAACCTCTCCAACTGC
YP_Etag 063	3943870	3944000	GCTTCGCCGTTTATCACATCGATTTACAGAGAAAGTAATCTCTGGCGGCAACGCCTTACACTGTACGCTGCTGTCACCCCATAATACCCACTTGGAGTTATATCACAATCTATAACATGCATTTATTATG
YP_Etag 064	3955098	3955198	CATCACAGCGCCTAACTTGTGGTGATGGGGCCTTTGTGGAAGAAGAACAACACCTAGTAATTAAAGCGATAACCCCACTGCGCGCCCTCTTGATTGATTT
YP_Etag 065	3965883	3965983	TCGAGCCAAACTTCATAACCTTGTTGTTGATAACTGCTCAGCAAATCCAGAACGGCATCGGGCACACAATCCGGGCGAGTCCCCACGCACAGGCCCACAA
YP_Etag 066	3994470	3994570	TTTTGATGCAGTATTGACCCGTGAACGCATGGGTAGCACCGGGATTGGCAGTGGTATCGCAATACCTCATGGCAAGTTGGAAGAGGATACACTGCGCGCG
YP_Etag 067	3998082	3998232	TATGATATCCCTTTCCGCATTACGTAAATCAGGAGTCAAGATGTTACGTGCTATCAAAACTAAACTCGCCCCTGCTGCCATTGGCCCTTATGTTCAAGGTGTCGATCTGGGCAGCATGATCATGACTTCCGGTCAGATCCCGATCGATCC

YP_Etag 068	4107295	4107445	TTCCTTTGCCAGCCAGGAGTACGGGGAAATACGGGCTTTATCGCGGCGTGGATAATTGATTGTCACACTGTGTAGTGGGTGACCATATTCATTGCAACGCAGCACAATCTGCTGGCTGCATTGCGGGTCTTGCGCGATACGTTCGTAGTG
YP_Etag 069	4108043	4108143	TTTTCATCCAACCAGAATTGGACAGAACTGCGGTAAAGCAACTGACTCTCTGCGCCCATATTATTGTTGGTGGTGCACAGTAAATAGGGTTTACTGGCGA
YP_Etag 070	4108256	4108406	ATGCCTGTTGGCAACGCGATAGCCAGTGGTTCGGCAAAGCGGTTACCACTCTGGTTGCGGTAAATGAGCAGTTGGCTGGTGGTGGCATAAATAAGGTCAATGGTACCGGAGCCATCAATATCTGCCAGATAGATCGCTAGTGGATTAAAT
YP_Etag 071	4109333	4109433	AAGCCATAGTTAAAGTGTGAGAAGGCGTCCTGACGGAAATTCCAGGATTTTCCTTTTATGATTACCGGCACTTGCTCCGCGATATTACTGTATTCGCCGT
YP_Etag 072	4112156	4112256	AAGCTTGATCGCCTTGGCTAATAATCAAGTCTAAGGTACGCATAAAGATCGCCAGCTTATATTGCATCGGATCTTGCATGGCAATAACATCCGGATCATC
YP_Etag 073(YP-3)	4125519	4125619	ATTGTACAAAAATCTCTGTTAGACTCAGACCTATGGCGTTCTCTATTTTATCCGTATAAGTCGTCGTATTACTACGTCGCCACGGCACTTTTCTGAACGA
YP_Etag 074	4189201	4189301	TCATCACCACGGCGAATAGCAACAATCACACAAGGAGCAATTTGCGGATAATAGCGCTCCCGACAGTGGTTACATAAGCTGGCCCATTCCGTCCGGCTGG
YP_Etag 075	4190854	4191004	CCATCCCGGTCTATGACACCTCCGGTCCCTATGGTGACCCACAAGCTAAACTGGATGTTCATAACGGGCTGCCTAAACTGCGTGCCGCTTGGGTCGCAGATCGCCAAGATACTGAAGCGCTGGCATCTGTCAGTTCCGGCTTTACCCAAC
YP_Etag 076	4439029	4439179	CCGTTATCGAGAGCAAAGCTAACGGATTGATTAGCTAATAGGTTGTCATGAGCATCGGTAACCAACGCTTGTGCAATGTTACCGGCTTTACCATTCGCAAGCTGGTTATCCAAGAACGACGTTAGCTTAAGCTGGGCTGTCGAAACATCG
YP_Etag 077	4442159	4442309	CACCCGAGACGGTATGCGTCAGTGTGGTACTGGCAGTACCATTAGCTCCTGTTCTTGCCTCTGTCGTACTTAATGTTGCCTGAGTATTAACCGCAAAGTTGATGAGCTGCCCGATAATCGGTTGGCCGTCGCCATCGGTTACTGTGGCTT
YP_Etag 078	4450113	4450263	CCTGTAATTGGCATGTCGATAGTTGCACGCTGACCAAAGTTGTCAGAAGCAGCGAGTACCGCAGGATTTGACGTCACTCCAGCTTATCTTTTTCACGTGGCCCAAGAGAGGAAGCCTGAGTATGATGAACCTGTCTTTCACGCATGTTGA
YP_Etag 079	4450281	4450431	GGTAACCATCATTAAATTCATACTCACGGCAAACCCGTGTCCAGGCAGCATAGGCGCTGACTATACGTTCATACTCTTCGGGGCGAAGATTATCTCTGGATAAGTTGTACAAATAGATGCCGAGAAGGCTTAGGCTCATCTCAACTCCTG
YP_Etag 080	4450664	4450764	AATTACATCAGGCTCATCCGGGCCGGGTTCTTCCAAGGCGTCAAATTGGCTTCTCAATAAATCGCTTGGCATAAAATGGCCGGAACGTGCTTGCAACCGC
YP_Etag 081	4451806	4451956	CATTGCCACCATTTTCAACGCCGATATGGGCAAAACGCTGCTGTACGGCACGTTGCTGGCAATCCCAACCGTGATTTTGGCAGGCCCGGTGTACGCCCGCTTTCTAAAAGGAATTGATAAGCCTGTTCCTGAAGGGTTGTATAACCCGAA
YP_Etag 082	4456200	4456300	AAAATAATGTTCATTCGGCGCGGTAATGGTTTATTTAACTGTTCTCACGAATAAACACGTCTAATTCCGCCAGTGGCTTTGCACCATCAATAGCATTGGC

YP_Etag 083	4462370	4462520	TTTATTCATAACGCGGTGCCTGTGGGTGAATGTCGATAGCCGCTAGTCAATCACAAGCCGGTAAATGCAAAATGTCAGAAGCCGTTCATAATTGTCATAAAGTTGAATAGTTAACCGAGATCACATTCCCGATAGGGAAATCATCGCTAG
YP_Etag 084	4462624	4462724	AACTTGCTCGTGGTGGCGGTCACCCTGATGGTGAGTGGTGTCGCGGTGCTGGGCTTCGAGCAGGCAAGCCGCTTACAAAAGCAGGTCCAGGAGCGGACAC
YP_Etag 085	4468034	4468184	TCAGCCCAGATGACCGCGGCGGTCTCTATCGGTATTGCCAGTTATACCGGCATGCCAGTATCGACCACTCAAGTGCTCTCTTCTGCTGTTGCCGGGACCATGTTGGTCGATGGCGGTGGCGTGCAGAGTAAAACGGTGAAGAGCATTATG
YP_Etag 086	4469634	4469784	TATTAGTGTAGGAAATGAAATGGAAAATAGCCTGATAATCTCGCATTTAAGCCTATCTTGCCGTTTGTTCAATACCGATTAATGTTAACGTCTCATTGGTATTGATTGTTATCACCGTATTTCTCGCGCTGATCACCGTGCTCAAACATT
YP_Etag 087	4472832	4472952	TATGCCTATTATCCTAATCCAGGTAGCTTCAGCCCAATATGGATTAATTACAGTTTCAGCGATAACCAACATCCCAGCAGATTAAATGATGGTGGCGGAGTATTAACACATGAGATCGGC
YP_Etag 088	4479982	4480082	CTCAGCAAGGCAGTGCGGCTTCAGTGGCATCGATGGCAGCCTATTTAGGATTAGACCTCACACTGCCACGCATTGCGCTGATCGTCAGTCTGCGTGAACA
YP_Etag 089	4480082	4480232	AGAGCACACTCAGCAGCGGGATCTGGTCGAAATATTAAGCAACCACAGCCGTGAAACATTGGTCACCTTACATGGGTTTGAGCGGGTTGTGGTGCTATTGCCGCTGAACCTTGCACACAGTGACGATCGTGAAGTGACGGCCAGAAAGGC
YP_Etag 090	4481162	4481262	ACTGGCACTGGCAGTTGCGACGATGCTGCTGACAGCGGTCGGGGTGTTTATCGATGTCGCGGTGATTACCGTTTCCCCAATCGCTCTGGCCATTGCGCAC
YP_Etag 091	4481507	4481607	TGATACTGCGCTGCCTGCCTTTGGTGCAGCAATGGCGGCACCTCTGGTCGCCATCTTCCTACTGGCATTACGCCCTATAGCTGGCATTGCCATTGACCCC
YP_Etag 092	4482938	4483088	TACAACCGGGGATTGAGATAGTCACCGAAGCGCTAAAATTAGCGGCTACCGTGCAAAATGCTGATTTAGTCATTACCGGCGAAGGGCGTATCGATAGCCAAACCATTTACGGCAAAACACCGGTCGGGGTGGCGCGAGTCGCGAAGCGTT
YP_Etag 093	4488099	4488249	GTCGCATTTGAGCCACCCGCTGAAATATAGATACCCGCACCCTTGGTCGCATGAGTACTATTAATGGTGTTATTCGCGCCCGTTAAGCTCCCTCCGGAACCATCCAGATAAATGCCATAGCTACCACTGCCACTGGTATTGAGGGTGGTA
YP_Etag 094	4491184	4491334	CGTTACGGTCTGGCAATAACCGCACTACCCTCTGGGGATTTTTACTGCTGGCCAACAAAGCAAGCCTGATAACCCAGGATCGCGCCTCAGCAGAGGAGCTGCAAGATCACACCTGTCAGGTGCTACCTCACCTCCCGGTGCAATAACAC
YP_Etag 095	4492530	4492630	TCCCGGATAAGACCAATTGTAGATCCAGCCCCGCCAGATTTCCGGGATCGGTTAAGGTCCCGACGACGGCCACCAGAGTAGAACCGTAGCGCATATCCGC
YP_Etag 096(YP-4)	4493379	4493529	CAAAAGGCCGTTGTAACTCGGCGGAGACTTTTTGGTTAATCGTTGGCTTCAAGCGATTCCAGTCAAAAGAAGAGACAAATATCACCACCGCCACCAATGACAACAGGATAAACCCACTGGCGCCCACCAATACTTTTCCGGTTCTTGTCA
YP_Etag 097	4595084	4595184	TTTTGCCACCCAGATAATGCAGTGAAATGATTCGAGTCTACCAACCCGACGATCTCAATGCCCTGATGCAACTGTGGCTAACCAGTACTATTGACGCGCA

*The *Y. pestis* CO92 strain was used as the reference (GenBank: NC_003143, 13-DEC-2020).
